# Exploring the Role of Autologous Fat Grafting in Implant-Based Breast Reconstruction: A Systematic Review of Complications and Aesthetic Results

**DOI:** 10.3390/jcm14124073

**Published:** 2025-06-09

**Authors:** Maximilian Vlad Muntean, Ioan Constantin Pop, Radu Alexandru Ilies, Annika Pelleter, Ioan Catalin Vlad, Patriciu Achimas-Cadariu

**Affiliations:** 1 Department of Plastic Surgery, “Prof. Dr. I. Chiricuță” Institute of Oncology, 400015 Cluj-Napoca, Romania; maximilian.muntean@iocn.ro; 2 Department of Plastic and Reconstructive Surgery, “Iuliu Hațieganu” University of Medicine and Pharmacy, 400012 Cluj-Napoca, Romania; 3 Faculty of Medicine, “Iuliu Hațieganu” University of Medicine and Pharmacy, 400012 Cluj-Napoca, Romania; annika.pelleter@lubos-kliniken.de; 4 Department of Surgical Oncology, “Prof. Dr. I. Chiricuță” Institute of Oncology, 400015 Cluj-Napoca, Romania; catalinvlad@elearn.umfcluj.ro (I.C.V.); pachimas@umfcluj.ro (P.A.-C.); 5 Department of Surgical Oncology and Gynecologic Oncology, “Iuliu Hațieganu” University of Medicine and Pharmacy, 400012 Cluj-Napoca, Romania

**Keywords:** aesthetic outcome, autologous fat grafting, breast implant, hybrid breast reconstruction, implant-based reconstruction, lipofilling, radiotherapy

## Abstract

**Background/Objectives**: Hybrid breast reconstruction (HBR), combining implant-based breast reconstruction (IBR) with autologous fat grafting (FG), has emerged as a promising solution to improve aesthetic outcomes and reduce complications, especially in irradiated patients. This study aims to systematically review current evidence on the outcomes of HBR with a focus on complication rates and aesthetic satisfaction. **Methods**: A systematic literature search was performed in March 2023 using PubMed and Embase databases. Studies were selected based on predefined PICOS criteria, including adult female patients undergoing IBR with FG. Sixteen studies met the inclusion criteria. Data on patient demographics, surgical techniques, fat grafting timing, complications, and aesthetic outcomes were extracted and analysed. **Results**: A total of 730 patients were included, with a mean follow-up of 20.23 months. The overall complication rate was 9%, with fat necrosis being the most common (2.7%). Capsular contracture occurred in 4.5% of cases. Immediate fat grafting during implant placement showed the lowest complication rate (8%) compared to delayed or staged procedures. Aesthetic satisfaction was high, with an average score of 4.4 out of 5 in five studies. Fat grafting improved skin quality and contour, especially in irradiated patients, and enabled the use of smaller implants. No oncological recurrences were reported. **Conclusions**: HBR is associated with high aesthetic satisfaction and a low rate of complications. Immediate fat grafting during IBR appears to be the safest and most effective approach. These findings support the wider adoption of HBR, though further high-quality studies are needed to validate long-term safety and outcomes.

## 1. Introduction

The expectations of patients undergoing breast reconstruction have grown in recent years. Creating a more natural-feeling breast without an autologous flap remains challenging. However, due to its less invasive approach, implant-based breast reconstruction (IBR) remains the preferred reconstruction method among patients, although it comes with higher risks involved, especially when patients underwent radiotherapy before. Since the current trend in prosthetic reconstruction has moved away from a submuscular technique to a muscle preserving prepectoral technique, a solution for visible implant rippling which comes more increasingly with the prepectoral approach had to be found. Autologous fat grafting (FG) added to an implant reconstruction not only helps with implant rippling but also offers the potential for significant improvement in the overall outcome [1]. In fact, it has been conclusively shown that autologous fat grafting in implant-based breast reconstruction enables results that could not be obtained by a use of an implant alone [2].

Beyond the improved aesthetical results, the technique also has medical indications, such as improving capsular contracture after IBR, helping wound healing issues following mastectomy and preventing the fibrotic effect radiation has on the implant-reconstructed breast [1,3].

Fat grafting in implant reconstruction provides the benefit of having a low rate of surgical complications compared to other procedures as well as establishing a high rate of surgeon and patient satisfaction [1]. However, the hybrid breast reconstruction has been a controversial topic in the past concerning its oncological safety: the two justifying main concerns were that, on the one hand, fat grafting might have had oncogenic potential, interfering with tumour surveillance; on the other hand, differences between post-fat grafting and neoplastic calcifications could not be distinguished from one another in radiographic imaging [2]. Since various studies have disproven these assumptions, fat grafting as an adjunct to IBR is a well-established technique with increasing popularity among plastic surgeons [4].

The aim of this study is to provide a systematic review of current evidence on the outcome of fat grafting in breast implant surgery, considering the complications and the aesthetic satisfaction of the patient and surgeon. In the following, the results of different studies with different fat grafting approaches that have investigated the complications as well as the aesthetic outcomes were analysed.

## 2. Materials and Methods

### 2.1. Literature and Search Strategy

For the inclusion of studies into the systematic review, PICOS eligibility criteria were used. The criteria are described in Table 1.

### 2.2. Information Source and Search Strategy

Articles were found using the databases PubMed and Embase for studies investigating the role of fat grafting in breast implant surgery. The word syntax used for the search included the following combination of terms: “fat grafting” AND “implant breast reconstruction”. The literature search was performed in March 2023.

### 2.3. Inclusion and Exclusion Criteria

After the search was performed, the inclusion criteria were evaluated based on their validity. Studies were deemed eligible if they met the following criteria:

- Adult female patient over 18 years;
- Studies published in English;
- The full text was available;
- Study type;
- Fat grafting secondary to breast implant reconstruction;
- Fat grafting during same procedure as implant reconstruction or before;
- Follow up period of at least 3 months;
- Study included outcome of complications or aesthetic satisfaction (or both);

Criteria for excluding the studies were as follows:

- Patients with chronic disease.;
- Studies which were published in a language other than English;
- Studies with no data on complications or aesthetic outcome;
- Single case reports;
- Studies reporting about breast reconstruction with exclusive fat grafting only;
- Studies that do not demonstrate original data, such as meta-analyses, systematic reviews, or discussions;
- In vivo studies on animals or in vitro studies.

### 2.4. Selection Process

**Figure 1:**
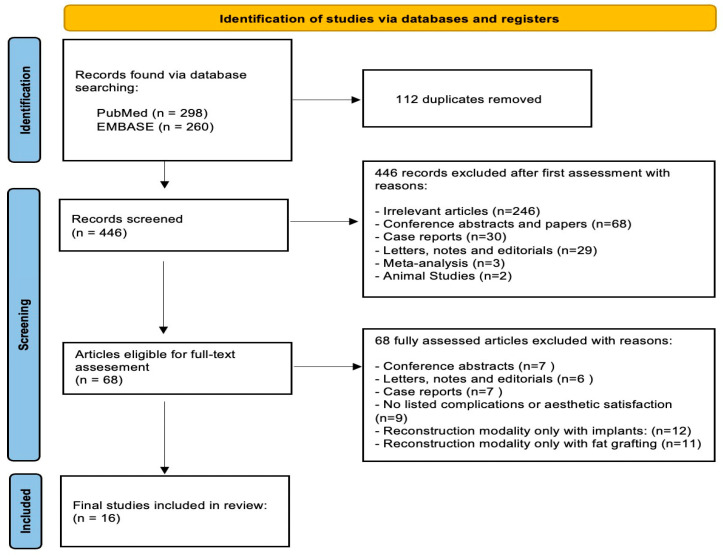
PRISMA diagram illustrating study identification and inclusion.

The articles obtained from the databases underwent initial duplicate screening. Subsequently, a preliminary screening was conducted by reviewing the titles and abstracts, applying the predefined inclusion and exclusion criteria. The full texts of the selected studies were then carefully examined to determine which ones met the criteria for inclusion in the review. Thereafter, a PRISMA flow diagram was created (as displayed in Figure 1). The included studies are shown in Table 2.

To minimise bias and enhance reproducibility, data extraction and study selection were performed independently by two reviewers. Any discrepancies between the reviewers were resolved through discussion and, when necessary, adjudicated by a third reviewer. This approach ensured consistent application of the inclusion and exclusion criteria and improved the reliability of the extracted data.

### 2.5. Data Extraction

Demographic information regarding the study type, the sample size the patients’ age, received radiotherapy or surgical procedure and fat grafting were extracted from the included studies and demonstrated in tables specifically designed for this systematic review.

Moreover, for each study, data related to complications, aesthetic score and main findings was extracted. Consequently, the collected data was compared and analysed, and a systematic review was performed. A meta-analysis was not performed.

### 2.6. Risk of Bias Assessment

A formal risk of bias assessment was performed using the Newcastle–Ottawa Scale (NOS), which is a widely accepted tool for evaluating the quality of non-randomised studies. NOS evaluates studies based on three domains: selection of participants, comparability of study groups, and ascertainment of outcomes. All case–control and retrospective cohort studies included in this review were assessed according to these criteria. Each study was scored on a scale from 0 to 9, with higher scores indicating lower risk of bias. For studies categorised as case series, the NOS was adapted in a limited and descriptive manner to provide an approximate sense of methodological robustness, although it is not formally designed for this study type. The NOS assessment was conducted independently by two reviewers, with discrepancies resolved through consensus.

## 3. Results

### 3.1. Study Selection

To begin with, 558 articles were retrieved for further evaluation, found via the databases PubMed and Embase. Out of those, 112 duplications were removed. Consequently, 446 articles were screened based on titles and abstract. The records were excluded after the first assessment due to being irrelevant, conference and abstract papers, case reports, meta-analysis or animal studies.

Subsequently, a full amount of 68 articles were retrieved for full-text assessment using the inclusion and exclusion criteria. A total of 52 were excluded because the reconstruction modality of the breasts is not a combination of implant insertion and fat grafting, or due to missing outcome data, or inappropriate study design. Eventually, 16 final studies were included in the current study. The study selection process is demonstrated in the PRISMA flow diagram (Figure 1).

### 3.2. Study Characteristics

The demographic characteristics of the included studies are shown in Table 2. The predominant study designs in this research include case series, case–control studies, and retrospective analyses. The included articles were published between 2011 and 2021. The studies were conducted in the United States, France, Italy, Spain, Israel, Belgium and Greece. The sample size ranged from 157 to as little as 3. The mean follow-up time was 20.23 months. All studies included assess a hybrid breast reconstruction with implants and fat grafting as an adjunct.

### 3.3. Patient Characteristics

A total of 730 patients with an average age of 48.5 years, ranging from 21 to 77, were assessed in the studies (Table 3). It could only be evaluated in 520 subjects whether radiotherapy was received or not. Therefore, out of 520 participants, 366 (70.83%) underwent radiotherapy after or prior to mastectomy. 100% of patients underwent lipofilling. Immediate fat grafting in the same procedure as implant placement was performed in 387 out of 730 patients (53%). Whereas only 11% received lipofilling in a secondary procedure, fat grafting prior to implant breast reconstruction was conducted in 263 out of 730 participants (36%).

### 3.4. Fat Grafting

All patients received fat grafting as an adjunct to implant breast reconstruction (Table 4). The mean volume grafted per session among all the 730 participants from the included study was 150.15 mL, ranging from 59.8 mL to as much as 313 mL. The participants underwent 1.84 sessions (1–5) of lipofilling on average.

The timing of fat grafting was conducted in three different reconstruction approaches. Immediate fat grafting in the same procedure as implant placement was performed in 356 out of 730 patients (49%). Whereas only 15% received lipofilling in a secondary procedure, fat grafting prior to implant breast reconstruction was conducted in 263 out of 730 participants (36%). A correlation between the timing of fat grafting and the number of lipofilling sessions needed and a correlation between the timing of fat grafting and the volume of fat injected per session could be made. Participants who received fat grafting prior to IBR needed the highest amount of lipofilling sessions on average (2.06 sessions) followed by the group receiving fat grafting as a secondary procedure after IBR (1.7 sessions) whereas patients undergoing IBR and fat grafting at the same time only needed a mean of 1.5 sessions of lipofilling. The mean volume of injected fat per session was also the highest in the group receiving FG prior to IBR with a mean volume of 189 mL (95.7–313 mL), also followed by the group undergoing lipofilling in a separate procedure (163 mL, ranging from 107 to 110). The smallest average volume per session (112 mL, ranging from 94 to 140) was injected in the group who underwent immediate fat grafting with IBR.

### 3.5. Complications

There was total of 72 (9%) complications, of which 51 (6.9%) were minor complications and 21 (3%) were major complications (Table 5). The most frequent complication was fat necrosis (n = 20, 2.7%), followed by seroma (n = 17, 2.3%), implant infection (n = 11, 1.5%), haematoma (n = 5, 0.6%), skin necrosis (n = 5, 0.06%), implant malposition (n = 2, 0.2%), and implant rupture (n = 1, 0.1%).

Due to incomplete reporting in some studies, the rate of capsular contracture was not clearly specified. Consequently, a separate analysis was conducted to examine this specific complication. Among the 16 studies included in the analysis, capsular contracture was mentioned in 9 of them, with a total of 24 cases reported out of 526 patients (4.5% incidence).

According to Calabrese et al. the complication rate in hybrid breast reconstruction is lower than in standard implant-based reconstructions without lipofilling, especially in capsular contractures [3]. A correlation between the timing of fat grafting in the reconstruction procedure and the complication rate could be made. Whereas participants who received IBR and FG at the same time only have a mean complication rate of 8% (29/356), patients who had lipofilling in a separate procedure prior to implant placement, had a slightly higher rate of 8.7% (23/263). Significantly higher, however, was the rate when fat grafting was performed in a secondary/tertiary approach after implant placement (12%). A correlation between the amount of injected fat per session and the complication rate could not be concluded.

### 3.6. Aesthetic Score

In 5 out of 16 studies, an aesthetic scoring system ranging from 1 to 5 was evaluated among the participants (Table 4) [13,14,15,16,17]. A score of 1 represents a poor aesthetic outcome, while a score of 3 indicates an average outcome, and a score of 5 signifies a superior aesthetic outcome [17]. The lowest average score given in a study by Serra-Renom et al. was 4 [16] whereas the highest evaluated scores were 4.8 in both articles published by Razzouk et al. [14,15]. Average aesthetic evaluation of the 5 studies was 4.4. Lakhiani et al. conducted a study in which he had patients separately evaluate the contour, volume and projection of the newly reconstructed breast separately on a scale from 1 to 5. It was concluded that, in comparison to the control groups using fat grafting or implant only, the hybrid breast reconstruction group had higher aesthetic scores regarding overall appearance, volume and contour, but not projection [17].

In terms of aesthetics, no significant difference between patient satisfaction and surgeon satisfaction was found [15]. A correlation between the fat grafting timing in the reconstruction procedure and the aesthetic outcome could not be made since in 4 of the 5 studies lipofilling was performed prior to prepectoral implant placement.

### 3.7. Risk of Bias Assessment

The Newcastle–Ottawa Scale (NOS) assessment showed that the case–control and retrospective cohort studies had moderate to high methodological quality, with scores between 6 and 8 out of 9 (Table 6). In contrast, the case series demonstrated lower methodological rigour, with scores ranging from 3 to 5, mainly due to lack of comparison groups and limited control of bias. These results indicate a moderate overall risk of bias in the included evidence.

## 4. Discussion

Autologous breast reconstruction is widely recognised as the gold standard for breast reconstruction when dealing with a breast that has been previously irradiated or is expected to undergo radiation therapy. Additionally, recent research has demonstrated that microsurgical tissue transfers lead to greater satisfaction with the overall outcome and the appearance of the breast. Nevertheless, implant-based breast reconstruction continues to be the preferred choice among patients, primarily due to its benefits of limited scarring, absence of complications at the donor site, shorter surgical duration, and the lack of necessity for specialised microsurgical skills [7,20].

Prosthetic reconstruction is typically not recommended for breasts that have been exposed to radiation therapy. Indeed, the use of implants in patients who have received radiation therapy is associated with a higher risk of unsatisfactory aesthetic results and various complications. These complications may include pain, capsular contracture, and thinning of the skin, which can lead to visible implants, implant deflation, and rupture [21]. Consequently, there is a growing tendency toward favouring hybrid breast reconstruction, combining the use of implants and autologous fat grafting, due to both aesthetic and medical considerations [5]. Combining fat grafting with implant reconstruction in patients who have received radiation therapy is proven to have a preventive effect on implant-related complications caused by radiotherapy, such as capsular contracture and skin thinning. Additionally, this approach has been associated with a notable increase in aesthetic satisfaction [1].

Despite lipofilling to the implant-based mastectomy reconstruction having been a controversial topic in the past, especially due to concerns about cancer recurrences, hybrid breast reconstruction has emerged as an important supplementary technique and is considered to be one of the greatest progresses in breast surgery in the past decade [1,22].

While it is considered a highly promising and advanced technique, there remains a scarcity of research on the subject.

### 4.1. Summary of Main Results

The findings of this systematic literature review corroborate previous studies’ claims regarding the relatively high satisfactory outcome and relatively low incidence of complications (9%). Calabrese et al. reports that, in comparison to the control group, which had a standard IBR, the HBR group had a lower complication rate, significantly in capsular contractures [3]. In this systematic review, the average incidence of capsular contracture was found to be relatively low (4.5%).

These results build on existing evidence of a study conducted by Ribuffo et al., showing that lipofilling exhibits protective properties, enabling the utilisation of implant-based breast reconstruction in the presence of radiotherapy, resulting in a significantly reduced incidence of complications [23]. The only reported complication explicitly associated with fat grafting is fat necrosis. Upadhyaya et al. stated that the incidence of fat necrosis becomes higher with an increasing amount of fat volume injected. Moreover, fat necrosis can mimic local recurrence on imaging and is often palpable, which may raise concerns of malignancy among patients and clinicians. This can lead to additional follow-up imaging, biopsies, or consultations, causing psychological distress and increased burden on healthcare resources [18]. Such a correlation could not be made in this review; however, based on the findings of a review by Spears et al., an association between the fat injection volume and the prevalence of fat necrosis was also made [24].

Patel et al. stated that the complication rate can be dependent on the timing of the fat grafting: In comparison to a delayed fat grafting in a tertiary procedure, immediate fat grafting during IBR comes with less complications (*p* = 0.0331), results in fewer additional surgeries (*p* < 0.001) and enables patients to finish the reconstruction more than one year earlier. (*p* < 0.001) [5]. The results of this systematic review support Patel’s theory indicating that the complication rate in patients undergoing fat grafting in a separate procedure after implant placement is significantly higher (12%) than in participants receiving IBR and FG in the same procedure at the same time (8%).

Moreover, the analysis revealed that fat grafting prior to implant-based breast reconstruction, the complication rate was determined to be 8.7%. As a result, it was concluded that immediate fat grafting in conjunction with IBR represents the safest approach to breast reconstruction. Further evidence on this topic could not be found anywhere else in the literature.

Another contributing factor that can reduce the incidence of complications associated with implants is the advantage of autologous fat grafting, allowing for the use of smaller implants [9,15]. Several studies reported that the use of fat grafting allowed for smaller implant sizes, and this may reflect anatomical limitations (e.g., in thin patients) rather than a desired aesthetic goal. Therefore, smaller implants should not be interpreted as having inherently superior outcomes. Future studies should incorporate patient preference and satisfaction more explicitly in relation to implant volume and shape.

The good-to-very good aesthetic outcome is described by multiple studies, the hybrid technique ensures a more natural appearance, a decrease in implant rippling, and better breast symmetry [9,17,19]. Five studies analysed in this review utilised a rating scale ranging from 1 to 5 to evaluate the aesthetic outcome. The average result obtained was 4.4, indicating a notably favourable and satisfactory outcome [13,14,15,16,17]. These results should also be considered when deciding between a simple implant reconstruction or a hybrid breast reconstruction: Lakhiani et al. concluded in his study that the aesthetics satisfaction rate is higher in IBR with fat grafting than IBR alone [17]. The positive outcomes are reinforced by the findings of Cogliandro et al., who demonstrated a significant improvement in BREAST-Q scores among patients who underwent lipofilling [10]. The importance of aesthetic appearance for patients is growing in tandem with the influence of social media, leading to a substantial rise in the expectations placed on plastic surgeons [1].

Further findings include that lipofilling as an adjunct to IBR is an important indication to prevent skin trophicity [15,16]. While Serra-Renom et al. highlighted the improved skin quality, including increased elasticity and hydration, associated with the use of lipofilling, Razzouk et al. and Sommeling et al. reported that autologous fat grafting helps the cutaneous and subcutaneous tissue form a thicker skin envelope enhancing the quality of mastectomy skin flaps [9,14,16].

Another important finding of medical significance by Cogliandro et al. is that the grafted fat ensures pain reduction in postmastectomy-pain syndrome [10]. This hypothesis could not be assessed in this review due to lack of information; however, a study conducted by Caviggioli et al. supports the assumption of Cogliandro et al., stating that the incidence of pain significantly decreased in patients receiving fat grafting [25].

Concerning oncological safety, which has been a heated topic discussed in the past, no recurrences were detected in the study by Ho Quok et al. [19].

In their study, Hammond et al. investigated the use of fat grafting (lipofilling) in implant-based breast reconstruction, a technique that combines long-term reconstructive benefits with aesthetic improvements by filling and shaping the breast tissue. While most reported complications were minor (fat necrosis and wound dehiscence), the occurrence of a major complication (red breast) suggests that, despite the benefits, there are associated risks. Minor complications like fat necrosis are common in fat grafting procedures but are generally manageable and reversible [6].

The study by Gronovich et al. explored a hybrid prepectoral direct-to-implant reconstruction technique combined with autologous fat grafting in immediate breast reconstruction. The authors demonstrated that this approach improves aesthetic outcomes by enhancing breast contour, reducing upper pole rippling and deflation, and achieving more natural-looking results. The study, which included 15 patients and 25 reconstructed breasts, found that the addition of autologous fat grafting to the procedure minimised the need for additional revisions and led to satisfactory results in all cases, with no major complications observed [8].

The study by Cigna et al. investigated the use of secondary autologous fat grafting (lipofilling) to correct asymmetries and contour deformities in patients who have undergone breast reconstruction with implants. The authors reviewed the outcomes of fat grafting in 20 patients who had nipple-sparing, skin-sparing, and skin-reducing mastectomies between 2008 and 2011. The results indicate that lipofilling is a safe and effective procedure, with no major complications occurring postoperatively and donor sites healing well without visible scars. The study also reported a significant improvement in aesthetic satisfaction, as assessed by both patients and independent plastic surgeons using the Visual Analog Scale (VAS). The average satisfaction scores increased from 5.2 preoperatively to 7.9 one month postoperatively and remained high at 7.2 after six months. The authors conclude that lipomodelling is a reliable, low-risk, and predictable solution for addressing contour deformities in breast reconstruction patients, providing an easy-to-perform and effective cosmetic improvement [11].

The study by Salgarello et al. explored the use of fat grafting followed by implant placement as a reconstructive option for irradiated breast cancer patients. The authors reviewed 16 cases of patients who received fat grafting followed by breast implant placement. The goal was to improve breast contour and reduce radiation-induced complications, which are common in postmastectomy patients. The study showed that fat grafting could improve the outcome of implant-based reconstructions in irradiated breasts, with a high patient satisfaction rate and no short-term complications. A Baker grade 1 capsule contracture was observed in all cases. The authors concluded that this approach may be a viable option for carefully selected patients, though they recommend larger studies with longer follow-up to further assess the long-term benefits and safety of the technique [12].

### 4.2. Applicability of Evidence

Overall, these findings suggest that incorporating lipofilling and utilising immediate fat grafting with implant-based breast reconstruction can lead to improved outcomes, including a high level of patient satisfaction, lower complication rates, and enhanced aesthetic results. It is important to consider each patient’s individual circumstances and preferences when making recommendations for breast reconstruction. Since the hybrid breast reconstruction is a relatively safe, less invasive approach, it is easily applicable in clinical practice, assuming the patient’s appliance and the patient being a non-smoker [1]. Fat grafting may have a potential benefit for surgeons by allowing them to overcome limitations in the muscle sparing prepectoral breast reconstruction, as the current trend is shifting away from the more invasive subpectoral technique.

Many surgeons tend to avoid the prepectoral approach in breast reconstruction due to the heightened risk of mastectomy flap necrosis and contour deformities. Nevertheless, fat grafting has the potential to improve not only the thickness of the flap but also its overall contour, providing a viable solution to address these issues [22].

Immediate fat grafting in prepectoral cases is technically feasible only in selected patients with thick and well-perfused mastectomy flaps. In such cases, the flap can accommodate small volumes of fat without compromising vascularity. However, in patients with thin flaps, this approach may not be possible or safe, introducing anatomical variability as a confounding factor [8,15]. This introduces anatomical variability and a potential confounder when comparing outcomes across studies.

In addition, it is important to consider that the surgical technique may involve multiple procedures, which can prolong the reconstruction process, making it more time-consuming and costly. From a psychological perspective, it is necessary to acknowledge that the longer the procedure takes, the longer it may take for the patient to emotionally embrace their new breasts [1].

Moreover, an important factor in practice is that one must consider that fat grafting cannot be conducted in very skinny patients and that the outcome of fat grafting, especially its remanence, can be unpredictable. Usually, however, a gradual loss of 20–30% of the volume acquired through fat transfer has been observed and the volume remains stable after 3 to 4 months unless the patient loses weight. Thus, it is mandatory to inform patients about potential revisionary lipofilling sessions [2,22].

Although quite a large amount of data was analysed in this systematic literature review, there is still very limited data available about the role of lipofilling in implant breast reconstruction, especially on aesthetic outcomes. Therefore, it is advisable to undertake further investigations and research on this topic.

While previous research has focused on complications, the results of our analysis and Patel et al. are the only ones in publicly accessible articles who analyse the correlation between timing of fat grafting and complication rate [5]. This correlation is of great importance considering that fat grafting is seen as one of the greatest advances in breast reconstructive surgery and we strongly recommend conducting more studies on this. Since we could only review three case–control studies comparing a group receiving IBR and FG with a control group only undergoing IBR, plastic surgeons should be encouraged to conduct more case–control studies like that so that more data can be retrieved on the role of fat grafting.

The evidence presented by Salgarello et al. suggests that fat grafting followed by implant placement could be a viable reconstructive option for irradiated breast cancer patients, offering significant improvements in breast contour and reducing radiation-induced complications [12]. However, the applicability of this evidence depends on several factors, including patient selection, the extent of radiation damage, and the feasibility of multiple fat grafting procedures. While the results are promising, particularly with high patient satisfaction and low complication rates, the study’s retrospective nature and relatively small sample size limit the generalizability of the findings.

Ultimately, recent research has revealed that fat grafting is not only about volume restoration, but also about the regenerative properties of adipose-derived stem cells. These cells may have the ability to improve tissue quality, particularly in irradiated or fibrotic areas. While fat resorption of approximately 20–30% is commonly observed within the first few months, it is increasingly recognised that lipofilling is not only a volumetric technique but also a regenerative one. Efforts to enhance graft viability through stem cell enrichment or supportive therapies are ongoing and may transform future clinical protocols [2,22,26,27].

### 4.3. Limitations of the Study

This systematic review has some limitations. Firstly, research was not comprehensive to all databases, but only to PubMed and Embase. Some articles on the role of fat grafting in breast implant surgery could not be retrieved due to no free access to literature.

Secondly, the included studies lack equivalent data that can be compared across different studies, especially in the case of aesthetic outcome, for which different scoring systems were applied, so we could only assess five studies in the end. In some studies, also assessing other reconstruction techniques than IBR with FG, it was not clarified which type of complications applied to what reconstruction modality. The incidence of capsular contracture was only demonstrated in nine studies.

Additionally, the included studies display heterogeneity in sample size, patient selection, and surgical protocols, which limits the generalizability of the findings. Most studies included relatively small cohorts, reflecting the current limited but growing clinical experience with hybrid breast reconstruction.

The technique of fat grafting—including harvesting method, processing system (e.g., centrifugation, filtration), and injection approach—was inconsistently reported across the studies. This variability introduces a significant confounder and limits reproducibility and standardisation of results.

Insufficient information was observed regarding the quality of mastectomy before and after the surgery, the type of implant used, and the positioning of the implant, making it difficult to compare the study outcomes at an advanced level.

Lastly, although a formal risk of bias assessment was performed using the Newcastle-Ottawa Scale, the overall methodological quality of the included studies remains moderate. Many were retrospective or descriptive case series, inherently prone to selection and reporting bias. The lack of randomised controlled trials and the heterogeneity in study design further reduce the level of evidence and limit the strength of conclusions that can be drawn.

## 5. Conclusions

All in all, this systematic literature review provides additional support to previous studies regarding the favourable outcomes and low incidence of complications associated with implant-based breast reconstruction combined with fat grafting.

Immediate fat grafting during reconstruction is recommended for better results and fewer complications. In comparison to simple implant breast reconstruction, fat grafting in addition to implant-based breast reconstruction has a decreased rate of complications and improved aesthetic outcome. The use of autologous fat grafting allows for smaller implants and lipofilling has potential benefits in preventing skin issues and reducing postmastectomy pain. No recurrences were observed in terms of oncological safety. Further research is needed to validate these findings, especially in areas with limited data.

## Figures and Tables

**Table 1:** PICOS criteria for inclusion of studies.

Parameters	Inclusion Criteria
Participants	Female participants, clinically healthy, aged ≥ 18 years
Intervention	Implant breast reconstruction with autologous fat grafting
Comparison	Implant breast reconstruction alone
Outcome	Complications, aesthetic satisfaction
Study design	Case series, case–control, retrospective

**Table 2:** Demographic characteristics of the included studies.

Authors	Study Title	Year	Country
Patel et al. [5]	The safety and efficacy of autologous fat grafting during second stage breast reconstruction	2021	United States
Hammond et al. [6]	Total envelope fat grafting: A novel approach in breast reconstruction	2015	United States
Stillaert et al. [7]	The Prepectoral, Hybrid Breast Reconstruction: The Synergy of Lipofilling and Breast Implants	2020	United States
Gronovich et al. [8]	Hybrid Prepectoral Direct-to-Implant and Autologous Fat Graft Simultaneously in Immediate Breast Reconstruction: A Single Surgeon’s Experience with 25 Breasts in 15 Consecutive Cases	2022	Israel
Sommeling et al. [9]	Composite breast reconstruction: Implant-based breast reconstruction with adjunctive lipofilling	2017	Belgium
Cogliandro et al. [10]	The Role of Lipofilling After Breast Reconstruction: Evaluation of Outcomes and Patient Satisfaction with BREAST-Q	2017	Italy
Cigna et al. [11]	Secondary lipofilling after breast reconstruction with implants	2016	Italy
Salgarello et al. [12]	Fat grafting and breast reconstruction with implant: Another option for irradiated breast cancer patients	2012	Greece
Sarfati et al. [13]	Adipose-tissue grafting to the post-mastectomy irradiated chest wall: Preparing the ground for implant reconstruction	2011	France
Razzouk et al. [14]	Skin trophicity improvement by mechanotherapy for lipofilling-based breast reconstruction postradiation therapy	2020	France
Razzouk et al. [15]	Breast Reconstruction Combining Lipofilling and Prepectoral Prosthesis after Radiotherapy	2020	France
Serra-Renom et al. [16]	Fat grafting in postmastectomy breast reconstruction with expanders and prostheses in patients who have received radiotherapy: Formation of new subcutaneous tissue	2010	Spain
Lakhiani et al. [17]	Maximizing aesthetic outcome in autologous breast reconstruction	2014	United States
Upadhyaya et al. [18]	Outcomes of Autologous Fat Grafting in Mastectomy Patients Following Breast Reconstruction	2018	United States

**Table 3:** Study characteristics and main outcomes on FG in IBR.

Author	Study Type	Patients (Mean Age)	RT (%)	Technique	Key Findings
Patel et al. [5]	Case–Control	157 (48.2)	31%	TE → IBR + FG (Immediate/Delayed)	Immediate FG reduces complications and surgical burden vs. delayed.
Hammond et al. [6]	Case Series	22 (47)	N/A	IBR + Immediate FG	Reliable technique with low complications.
Stillaert et al. [7]	Case Series	33 (42)	3%	TE → FG → Prepectoral IBR	Improves outcomes and reduces tissue strain.
Gronovich et al. [8]	Case Series	15 (44)	27%	Immediate IBR + FG	Hybrid IBR improves contour, reduces rippling and reoperation.
Sommeling et al. [9]	Case Series	15 (46)	40%	TE → FG → Prepectoral IBR	Improved aesthetics, requires multiple stages.
Cogliandro et al. [10]	Case–Control	46 (41)	74%	IBR → Delayed FG	FG improves aesthetic and psychological outcomes post-RT.
Cigna et al. [11]	Case Series	20 (65)	0%	IBR → Delayed FG	FG effective for contour correction with minimal morbidity.
Salgarello et al. [12]	Case Series	16 (41)	100%	FG → IBR	Pre-implant FG mitigates RT-related implant issues.
Sarfati et al. [13]	Case Series	28 (45)	100%	FG → IBR	>80% good/very good results; no capsular contracture.
Razzouk et al. [14]	Retrospective	32 (50.6)	100%	FG → Prepectoral IBR	FG improves flap thickness and skin quality.
Razzouk et al. [15]	Retrospective	136 (52)	100%	FG → Prepectoral IBR	Higher FG volume → smaller implants, improved skin trophicity.
Serra-Renom et al. [16]	Case Series	65 (65)	100%	TE + FG → IBR + FG	FG improves skin quality, prevents contracture.
Lakhiani et al. [17]	Retrospective	24 (N/A)	N/A	FG → IBR	Higher aesthetic scores in hybrid group.
Upadhyaya et al. [18]	Retrospective	171 (50.5)	N/A	TE → IBR + FG	Fat necrosis risk increases with higher volume (10.5%).
Ho Quoc et al. [19]	Case Series	18 (43.8)	N/A	IBR → FG	No recurrence; minor complications only.
Calabrese et al. [3]	Case–Control	84 FG + IBR, 130 IBR	26%	TE → IBR ± FG	FG group had lower contracture rate and better aesthetics.

**Table 4:** Details of FG in IBR and aesthetic outcomes.

Author	Study Type	Patients (Mean Age)	Mean Volume (mL)	Fat Grafting Sessions (Mean, Range)	Aesthetic Score
Patel et al. [5]	Case–Control	157 (48.2)	94	2.7 (1–5)	N/A
Hammond et al. [6]	Case Series	22 (47)	134	1.4 (1–2)	N/A
Stillaert et al. [7]	Case Series	33 (42)	262	2.7 (1–5)	N/A
Gronovich et al. [8]	Case Series	15 (44)	59.8	1	N/A
Sommeling et al. [9]	Case Series	15 (46)	313	3.2 (2–5)	N/A
Cogliandro et al. [10]	Case–Control	46 (41)	110	2.2 (1–3)	N/A
Cigna et al. [11]	Case Series	20 (65)	N/A	1	N/A
Salgarello et al. [12]	Case Series	16 (41)	95.7	2.4 (1–4)	N/A
Sarfati et al. [13]	Case Series	28 (45)	115	2 (1–3)	4.5 (3.5–5)
Razzouk et al. [14]	Retrospective	32 (50.6)	151	1.15 (1–3)	4.8
Razzouk et al. [15]	Retrospective	136 (52)	220	1.6 (1–3)	4.8
Serra-Renom et al. [16]	Case Series	65 (65)	140	2.4 (1–4)	4
Lakhiani et al. [17]	Retrospective	24 (N/A)	168.6	1.4 (1–3)	4
Upadhyaya et al. [18]	Retrospective	171 (50.5)	132	1.18 (1–3)	N/A
Ho Quoc et al. [19]	Case Series	18 (43.8)	107	1.3	N/A
Calabrese et al. [3]	Case–Control	84 FG + IBR, 130 IBR	88	N/A	N/A

**Table 5:** Overview of reported complications on FG in the selected studies.

Author	Study Type	Patients (Mean Age)	Total Complications	Minor Complications	Major Complications
Patel et al. [5]	Case–Control	157 (48.2)	20	13 (4 infections, 6 dehiscence, 4 seromas, 1 fat necrosis, 2 implant malposition)	7 (4 infections, 3 skin necrosis)
Hammond et al. [6]	Case Series	22 (47)	4	3 (2 fat necrosis, 1 dehiscence)	1 (1 red breast)
Stillaert et al. [7]	Case Series	33 (42)	4	1 (1 haematoma)	3 (1 TE infection, 2 implant infections)
Gronovich et al. [8]	Case Series	15 (44)	5	3 (2 seromas, 1 dehiscence)	2 (2 infections)
Sommeling et al. [9]	Case Series	15 (46)	1	0	1 (1 implant infection with fat necrosis)
Cogliandro et al. [10]	Case–Control	46 (41)	2	0	2 (1 infection, 1 implant rupture)
Cigna et al. [11]	Case Series	20 (65)	1	1 (1 fat necrosis)	0
Salgarello et al. [12]	Case Series	16 (41)	0	0	0
Sarfati et al. [13]	Case Series	28 (45)	4	3 (3 seromas)	1 (1 severe seroma)
Razzouk et al. [14]	Retrospective	32 (50.6)	5	4 (4 cystic fat necrosis)	1 (1 implant infection)
Razzouk et al. [15]	Retrospective	136 (52)	10	7 (7 cystic seromas)	3 (1 implant infection, 2 skin necrosis)
Serra-Renom et al. [16]	Case Series	65 (65)	0	0	0
Lakhiani et al. [17]	Retrospective	24 (N/A)	0	0	0
Upadhyaya et al. [18]	Retrospective	171 (50.5)	18	18 (12 fat necrosis in IBR, 6 in autologous)	0
Ho Quoc et al. [19]	Case Series	18 (43.8)	4	4 (4 hematomas)	0
Calabrese et al. [3]	Case–Control	84 FG + IBR, 130 IBR	3	2 (2 seromas)	1 (1 implant infection)

**Table 6:** Newcastle-Ottawa Scale (NOS) scores for included studies.

Author	Study Type	Selection (4)	Comparability (2)	Outcome (3)	Total Score (9)
Patel et al. [5]	Case–Control	4	1	2	7
Cogliandro et al. [10]	Case–Control	3	1	2	6
Calabrese et al. [3]	Case–Control	4	2	2	8
Lakhiani et al. [17]	Retrospective Cohort	3	1	2	6
Upadhyaya et al. [18]	Retrospective Cohort	3	1	2	6
Razzouk et al. [14]	Retrospective Cohort	3	1	2	6
Razzouk et al. [15]	Retrospective Cohort	3	2	2	7
Hammond et al. [6]	Case Series	2	0	1	3
Stillaert et al. [7]	Case Series	2	0	1	3
Gronovich et al. [8]	Case Series	2	0	1	3
Sommeling et al. [9]	Case Series	2	0	1	3
Cigna et al. [11]	Case Series	2	0	1	3
Salgarello et al. [12]	Case Series	3	0	2	5
Sarfati et al. [13]	Case Series	3	0	2	5
Serra-Renom et al. [16]	Case Series	3	0	2	5
Ho Quoc et al. [19]	Case Series	2	0	1	3

## Data Availability

Data sharing is not applicable to this review as no new data were generated.

## Abbreviations

The following abbreviations are used in this manuscript:

IBR	Implant based breast reconstruction
HBR	Hybrid breast reconstruction
FG	Fat Grafting
RT	Radiotherapy
TE	Tissue expander
